# Generation of time-synchronized two-color X-ray free-electron laser pulses using phase shifters

**DOI:** 10.1038/s41598-023-39322-z

**Published:** 2023-08-23

**Authors:** Myung-Hoon Cho, Teyoun Kang, Haeryong Yang, Gyujin Kim, Seong-Hoon Kwon, Kook-Jin Moon, Inhyuk Nam, Chang-Ki Min, Hoon Heo, Changbum Kim, Heung-Sik Kang, Chi Hyun Shim

**Affiliations:** 1 Pohang Accelerator Laboratory, Pohang University of Science and Technology, Pohang, 37673 Korea; 2 Department of Physics and Earth Science, Korea Science Academy of KAIST, Busan, 47162 Korea

**Keywords:** Free-electron lasers, X-rays

## Abstract

To optimize the intensity of X-ray free-electron lasers (XFELs), phase shifters, oriented *in phase* with the phases of the XFEL pulse and electron beam, are typically installed at undulator lines. Although a π-offset between the phases (i.e., an “out-of-phase” configuration) can suppress the XFEL intensity at resonant frequencies, it can also generate a side-band spectrum, which results in a two-color XFEL pulse; the dynamics of such a pulse can be described using the spontaneous radiation or low gain theory. This attributes of this two-color XFEL pulse can be amplified (log-scale amplification) through an undulator line with out-of-phase phase shifters. In this study, the features of two-color XFEL pulses were evaluated through theory, simulations and experiments performed at Pohang Accelerator Laboratory X-ray Free Electron Laser. The XFEL gain slope and energy separation between the two-color spectral peaks were consistent through theoretical expectation, and the results of simulation and experiment. The experimentally determined two-color XFEL pulse energy was 250 μJ at a photon energy of 12.38 keV with a separation of 60 eV.

## Introduction

X-ray free-electron lasers (XFELs) that emit with two simultaneous colors are powerful tools for investigating the fundamental properties of materials in biology, femto-chemistry, condensed matter physics, and many other fields. Two-color XFEL pulses are widely used in pump-probe spectroscopy, in which the XFEL pulses are split spectrally and temporally into two pulses. Tunability of the time separation between the pulses enables time-resolved studies, and two selected spectra can be used to target various atomic states of sample materials by exciting and exploring different resonances^1–3^. Such a time delay between two-color beams can be attributed to the methods employed for generating two-color XFEL pulses with distinct resonance conditions for each color beam. In these schemes, two different resonance conditions are satisfied by manipulating the electron beam^4–8^, which is split in time and energy, or by tuning the undulators^9–13^ via chicane insertion, which results in a time-split FEL spectrum. In crystallography experiments using time-split two-color XFEL pulses, the target samples may be damaged by the strong intensity of the first XFEL pulse, leading to undesirable results from the second XFEL pulse due to the damaged samples. This drawback can be mitigated by using time-synchronized two-color XFEL pulses, which are attractive for various applications, such as multiple wavelength anomalous dispersion^14^ and multicolor imaging^15^, and can help overcome the aforementioned issue.

The undulator lines in XFEL facilities contain phase shifters, which can be considered as mini-chicanes and are generally located at the intersection between the undulator segments. These phase shifters can delay the electron beam to control its phase and are thus used to match the phases of the electron beam and the XFEL pulse before entering the next undulator unit. For example, the phase shifters installed in Pohang Accelerator Laboratory X-ray Free Electron Laser (PAL-XFEL) can induce a delay of up to tens of X-ray wavelengths; however, they typically operate within one or two wavelengths^16^. In normal FEL optimization, phase-matching between the FEL radiation and the electron beam is realized by scanning the gap of the phase shifters installed in the undulator line; this process is applied to identify the beam-based in-phase condition. In the out-of-phase condition, in which the electron beam is out-of-phase by π from the in-phase condition, phase mismatching occurs between the electron beam and the XFEL pulse, and the FEL intensity at the resonant frequency becomes fully suppressed^16^. However, the side spectrum can survive and grow along the undulator line, resulting in time-synchronized two-color XFEL pulses. Although this phenomenon has been discussed in other studies on phase-matching strategies^17,18^, the out-of-phase condition is seldom explored, because it does not maximize the XFEL intensity at the resonance frequency. Notably, unlike other methods, this out-of-phase condition is advantageous for generating time-synchronized two-color XFEL pulses, and thus, warrants a detailed investigation.

In this study, we examined the out-of-phase condition of phase shifters and successfully generated and amplified time-synchronized two-color XFEL pulses using the phase shifters installed at the PAL-XFEL undulator line; Both simulation and experimental investigations were conducted to unravel the features and merits of the out-of-phase condition of the phase shifters. Herein, we presented a theoretical framework and explained the low gain theory to elucidate the mechanism underlying the generation of two-color spontaneous radiation. We examined the FEL beam dynamics by comparing the one-dimensional (1D) FEL simulation results obtained under the in-phase and out-of-phase conditions to analyze the change in the dynamics of the electron beam and XFEL pulse within the high gain regime. Further, we analyzed the feasibility of amplifying the intensity of the time-synchronized two-color XFEL pulse under the out-of-phase condition of the phase shifters. Finally, we experimentally generated time-synchronized two-color XFEL pulses and observed their saturation.

## Generation mechanism of two-color XFEL pulses

### Theoretical framework

**Figure 1:**
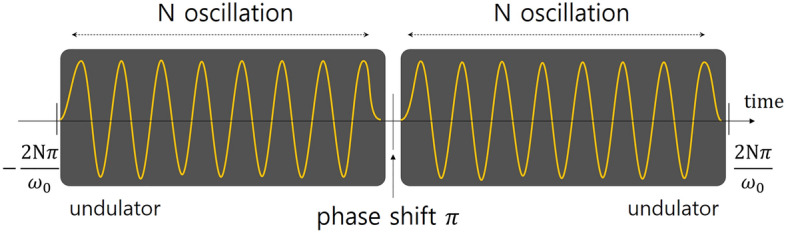
Schematic view of two-color radiation with a ${\varvec{\uppi}}$ phase-shift. The yellow lines indicate the electric field or electron motion following the undulators (the dark region).

First, we show the theoretical framework for two-color radiation under an out-of-phase condition. We consider two undulators, one phase shifter, and one electron. As the electron propagates through the undulator, well-defined sinusoidal waves are generated. The number of radiation wavelengths is equal to the number of undulator periods (N). When the electron traverses the phase shifter, it is delayed by half of a radiation wavelength (out-of-phase setting) and then enters the second undulator. In this case, 2N radiation wavelengths are generated, although the wave phase shows discontinuity at the intersection between undulators as illustrated in Fig. 1. If we set the time as zero at the center of the total radiation (when the electron is set to be out-of-phase), then the radiation can be described as:

1 $$f\left(t\right)=\left\{\begin{array}{c}-sin\left({\omega }_{0}t\right), -\frac{2\pi }{{\omega }_{0}}N\le t<0\\ sin\left({\omega }_{0}t\right), 0\le t<\frac{2\pi }{{\omega }_{0}}N,\end{array}\right.$$

where ${\omega }_{0}$ is the radiation frequency. Equation (1) can be reformulated into a single equation, which interestingly shows two-color radiation modes, as:

2 $$f\left(t\right)=\frac{\mathrm{cos}\left(\left(1+\frac{1}{2N}\right){\omega }_{0}t\right)-\mathrm{cos}\left(\left(1-\frac{1}{2N}\right){\omega }_{0}t\right)}{2\mathrm{sin}\left(\frac{{\omega }_{0}\left|t\right|}{2N}\right)}, -\frac{2\pi }{{\omega }_{0}}N\le t<\frac{2\pi }{{\omega }_{0}}N.$$

The numerator in Eq. (2) indicates that sequential waves, including those with a π shift, can be considered as the sum of two frequency modes, and each frequency is biased as $\pm {\omega }_{0}/2N$. This simple frequency definition is consistent with the analytical result obtained using the low gain theory as discussed in the following section.

### Low gain theory

Here, we develop a detailed analytical expression for describing two-color spontaneous radiation using the low gain theory. Based on the 1D Maxwell–Klimontovich equation, the electric field in the Fourier domain can be expressed as^19^:

3 $${E}_{\nu }\left(z\right)=\oint \frac{d\mu }{2\pi i}\frac{{e}^{-i\mu 2\rho {k}_{u}z}}{D\left(\mu \right)}\left[{E}_{\nu }\left(0\right)+\frac{i{n}_{e}}{2{\mathrm{k}}_{\mathrm{u}}\rho }\frac{eK\left[JJ\right]}{4{\epsilon }_{0}{\gamma }_{r}}\frac{1}{{N}_{\lambda }}\sum_{j=1}^{{N}_{e}}\frac{{e}^{-i\nu {\theta }_{j}\left(0\right)}}{{\eta }_{j}\left(0\right)/\rho -\mu }\right],$$

where $\nu \equiv 1+\left(\omega -{\omega }_{r}\right)/{\omega }_{r}=1+\Delta \nu$ is the relative radiation frequency, ${\epsilon }_{0}$ is the vacuum permittivity, ${\omega }_{r}$ is the resonant frequency, $\rho$ is the Pierce parameter, ${\gamma }_{r}$ is the resonant Lorentz factor of an electron, ${N}_{\lambda }$ is the number of electrons in a single wavelength, $K\left(=\frac{e{B}_{0}}{mc{k}_{u}}\right)$ is the undulator parameter ($e$ is the electron charge, ${B}_{0}$ is the undulator magnetic field, $m$ is the electron mass, and $c$ is the light velocity), ${k}_{u}$ is the undulator wavenumber, $\left[JJ\right]\equiv {J}_{0}\left(\frac{{K}^{2}}{4+2{K}^{2}}\right)-{J}_{1}\left(\frac{{K}^{2}}{4+2{K}^{2}}\right)$ is a harmonic factor, ${E}_{\nu }\left(0\right)$ is the initial electric-field amplitude in the Fourier domain, ${\theta }_{j}$ is the j-th electron phase, and ${n}_{e}$ is the electron beam density. The dispersion function is defined as:

4 $$D\left(\mu \right)=\mu -\frac{\Delta \nu }{2\rho }-\int d\eta \frac{V\left(\eta \right)}{ {\left(\frac{\eta }{\rho }-\mu \right)}^{2}}$$

where $V\left(\eta \right)$ is the electron distribution function. Equation (3) can be further modified using the residue theorem with the singularity condition of $D\left(\mu \right)=0$, which yields three solutions: ${\mu }_{1},{\mu }_{2},{\mathrm{and} \mu }_{3}$. Then, Eq. (3) can be rewritten as:

5 $${E}_{\nu }\left(z\right)=\left(\frac{{e}^{-2i{\mu }_{1}\rho {k}_{u}z}}{{D}{\prime}\left({\mu }_{1}\right)}+\frac{{e}^{-2i{\mu }_{2}\rho {k}_{u}z}}{{D}{\prime}\left({\mu }_{2}\right)}+\frac{{e}^{-2i{\mu }_{3}\rho {k}_{u}z}}{{D}{\prime}\left({\mu }_{3}\right)}\right){E}_{\nu }\left(0\right)+\frac{i{n}_{e}}{2{\mathrm{k}}_{\mathrm{u}}\rho }\frac{eK\left[JJ\right]}{4{\epsilon }_{0}{\gamma }_{r}}\frac{1}{{N}_{\lambda }}\sum_{k=1}^{3}\frac{{e}^{-2i{\mu }_{k}\rho {k}_{u}z}}{{D}{\prime}\left({\mu }_{k}\right)}\sum_{j=1}^{{N}_{e}}\frac{{e}^{-i\nu {\theta }_{j}}}{\frac{{\eta }_{j}}{\rho }-{\mu }_{k}}$$

According to Eq. (2), $\Delta \nu$ ≈ $1/2N$ $\ll 1$ for a normal undulator. For this assumption and a delta-function-like $V\left(\eta \right)$, the solutions of Eq. (4) are ${\mu }_{1}\cong\Delta \nu /2\rho , {\mu }_{2}={\mu }_{3}^{*}\sim 0$, which result in low gain or spontaneous radiation emission. Equation (5) is simplified as:

6 $${E}_{\nu }\left(z\right)={e}^{-i\Delta \nu {k}_{u}z}{E}_{\nu }\left(0\right)-\frac{i{n}_{e}}{2{\mathrm{k}}_{\mathrm{u}}\rho }\frac{eK\left[JJ\right]}{4{\epsilon }_{0}{\gamma }_{r}}\frac{1}{{N}_{\lambda }}\sum_{j=1}^{{N}_{e}}{e}^{-i\nu {\theta }_{j}}\frac{{e}^{-2i{\eta }_{j}{k}_{u}z}-{e}^{-i\Delta \nu {k}_{u}z}}{{\eta }_{j}-\frac{\Delta \nu }{2}}.$$

Now, we consider a simple case of two undulators, one phase shifter, and one electron beam. After the first undulator, Eq. (6) becomes

7 $${E}_{\nu 1}\left({L}_{u}\right)=-\frac{i{n}_{e}}{2{\mathrm{k}}_{\mathrm{u}}\rho }\frac{eK\left[JJ\right]}{4{\epsilon }_{0}{\gamma }_{r}}\frac{1}{{N}_{\lambda }}\sum_{j=1}^{{N}_{e}}{e}^{-i\nu {\theta }_{j}}\frac{{e}^{-2i{\eta }_{j}{k}_{u}{L}_{u}}-{e}^{-i\Delta \nu {k}_{u}{L}_{u}}}{{\eta }_{j}-\frac{\Delta \nu }{2}},$$

where ${L}_{u}$ is the undulator length, and ${E}_{\nu }\left(0\right)=0$ (assumption). For a phase shifter with a phase $\phi$, the final electric field description after the second undulator can be expressed by

8 $${E}_{\nu 2}\left({L}_{u}\right)=-\frac{i{n}_{e}}{2{\mathrm{k}}_{\mathrm{u}}\rho }\frac{eK\left[JJ\right]}{4{\epsilon }_{0}{\gamma }_{r}}\frac{1}{{N}_{\lambda }}{e}^{-i\Delta \nu {k}_{u}{L}_{u}}\left[\left({e}^{-i\Delta \nu {k}_{u}{L}_{u}}+{e}^{-i\nu \phi }\right)\sum_{j=1}^{{N}_{e}}{e}^{-i\nu {\theta }_{j}}\frac{{e}^{-2i\left({\eta }_{j}-\frac{\Delta \nu }{2}\right){k}_{u}{L}_{u}}-1}{{\eta }_{j}-\frac{\Delta \nu }{2}}\right].$$

Finally, we can calculate the power spectral density as:

9 $$\frac{dP}{d\omega }=\frac{{\epsilon }_{0}}{\pi c}\frac{{A}_{tr}{\lambda }_{r}^{2}}{T}\langle {\left|{E}_{\nu 2}\left({L}_{u}\right)\right|}^{2}\rangle =\frac{2{\epsilon }_{0}}{\pi c}\frac{{A}_{tr}{\lambda }_{1}^{2}}{T}S\left(\Delta \nu ,\phi \right){\left|{E}_{{\nu }_{1}}\left({L}_{u}\right)\right|}^{2},$$

where ${A}_{tr}$ is the cross-sectional area of the electron beam, ${\lambda }_{r}$ is the resonant wavelength, and $T$ is the electron beam duration. Here, the functions in Eq. (9) are defined as

10 $${\left|{E}_{{\nu }_{1}}\left({L}_{u}\right)\right|}^{2}\cong {\left(\frac{{n}_{e}}{2{\mathrm{k}}_{\mathrm{u}}\rho }\frac{eK\left[JJ\right]}{4{\epsilon }_{0}{\gamma }_{r}}\frac{1}{{N}_{\lambda }}\right)}^{2}{N}_{e}\int d\eta V\left(\eta \right)\frac{{\mathrm{sin}}^{2}\left[\left(\eta -\frac{\Delta \nu }{2}\right){k}_{u}{L}_{u}\right]}{{\left(\eta -\frac{\Delta \nu }{2}\right)}^{2}},$$

11 $$S\left(\Delta \nu ,\phi \right)=1+\mathrm{cos}\left[\phi +\Delta \nu \left(\phi -2\pi {N}_{u}\right)\right].$$

**Figure 2:**
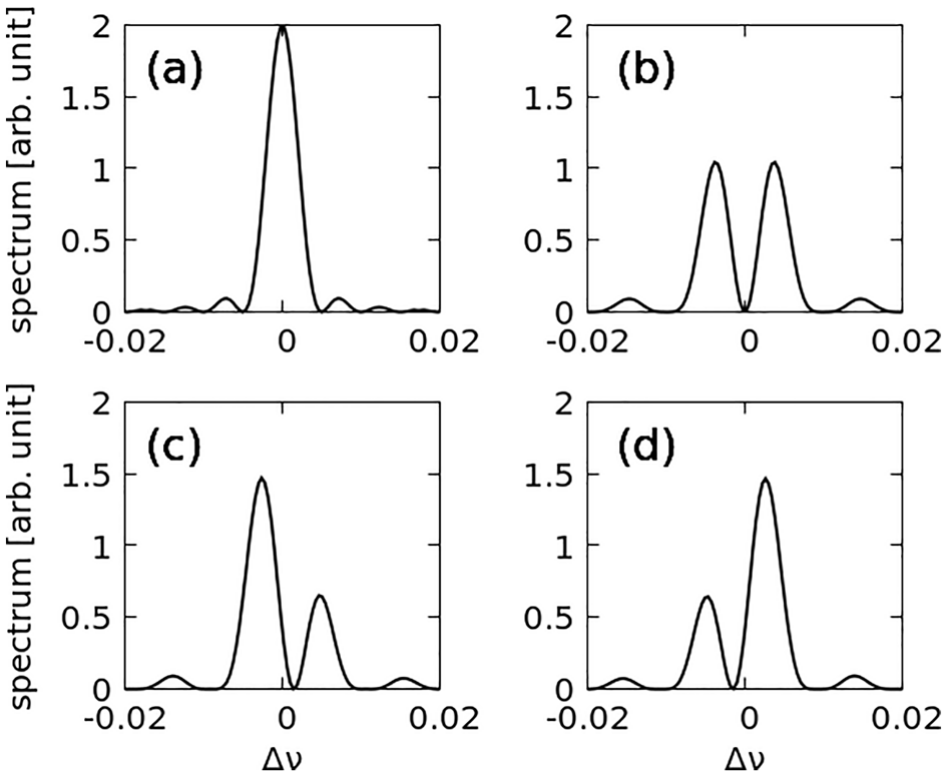
Spectra of spontaneous radiation (Eq. (9)) for different phases: (**a**) $\phi =0$, (**b**) $\phi =\pi$, (**c**) $\phi =-0.7\pi$, and (**d**) $\phi =0.7\pi$. Here, two undulators and one phase shifter are considered.

At $\phi =\uppi$, $S\left(\Delta \nu ,\phi =\pi \right)$ results in a maximum at $\Delta \nu =\pm 1/(2{N}_{u}-1)\approx \pm 1/2{N}_{u}$, which is the same as that presented in the previous section. Figure 2 shows some examples of ${\left|{E}_{\nu 2}\left({L}_{u}\right)\right|}^{2}$ at different $\phi$ values, and all the cases yield two-color spectra. In Fig. 2b, the two-color spectrum obtained at $\phi =\uppi$ is symmetric along $\Delta \nu$, because ${\left|{E}_{{\nu }_{1}}\left({L}_{u}\right)\right|}^{2}$ and $S\left(\Delta \nu ,\pi \right)$ are symmetric functions for $V\left(\eta \right)=\delta \left(\eta \right)$. Figure 2c,d show asymmetric two-color features. As the phase $\phi$ shifts in the positive direction, the spectrum peaks shift to high energies, and the intensity of the peaks in the side spectrum, obtained at low energies, increases. Conversely, when the phase $\phi$ shifts in the negative direction, the peaks in the spectrum shift to low energies, and the intensity of the peaks in the side spectrum, obtained at high energies, increases. As expected, for $\phi =\uppi$, two identical peaks are observed. In the example, we used ${N}_{u}=100$, and the peaks in the two-color spectrum at $\phi =\uppi$ are located around $\pm 1/2{N}_{u}$ (exactly, $\pm 0.372/{N}_{u}$). However, in reality, a possibility of asymmetry in ${\left|{E}_{{\nu }_{1}}\left({L}_{u}\right)\right|}^{2}$ exists, even for $\phi =\uppi$; this asymmetry originates from the nonideal energy distributions of the electron beam and undulator K along the length. The asymmetry of ${\left|{E}_{{\nu }_{1}}\left({L}_{u}\right)\right|}^{2}$ can violate the balance between two-color spectral peaks even at $\phi =\uppi$. Therefore, to obtain balanced two-color spectral peaks, we have to identify the optimal $\phi$ around $\phi =\uppi$ as discussed in the next section. This expected two-color feature was also discussed by Li and Pflueger^18^.

## Simulation results

**Figure 3:**
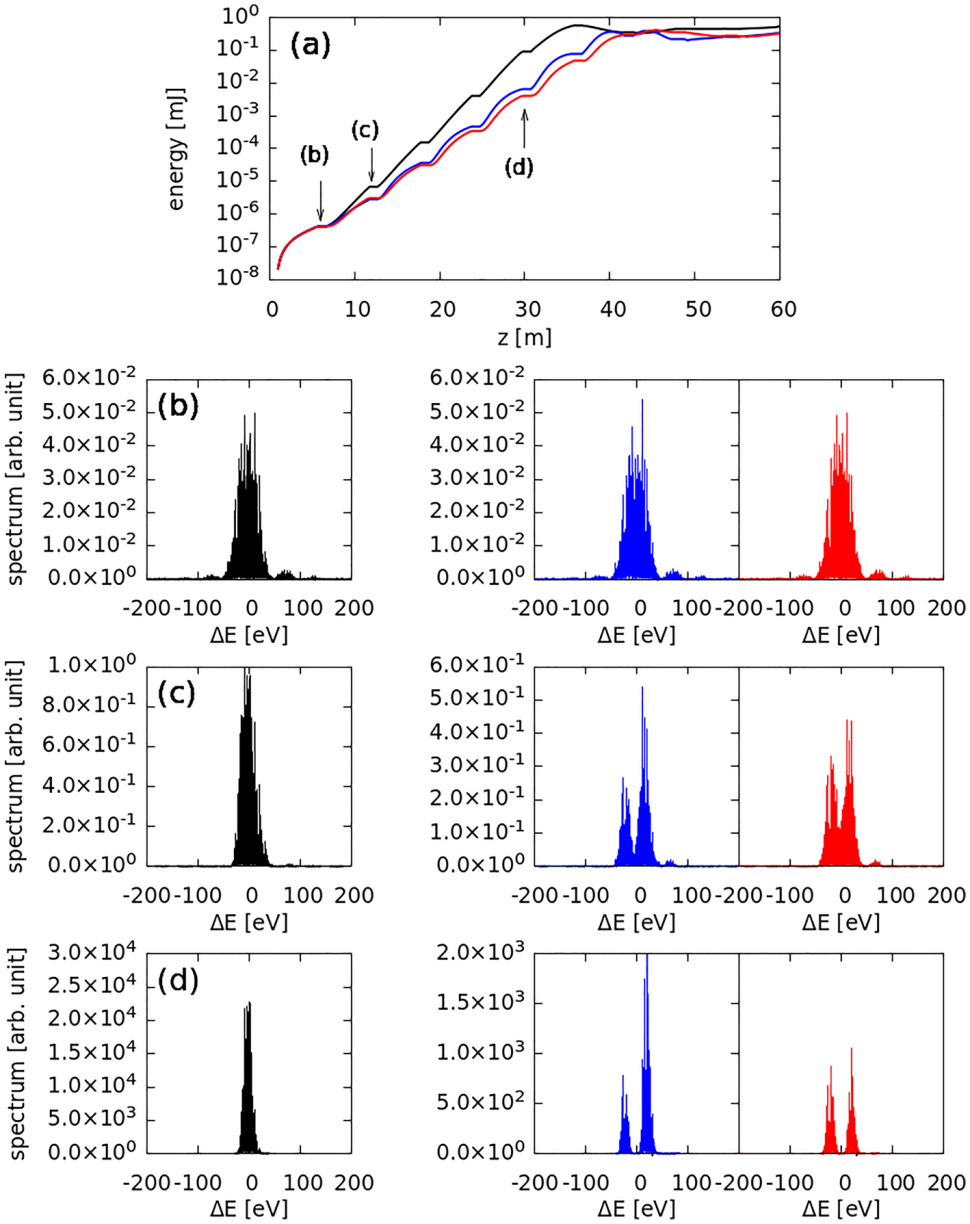
1D time-dependent FEL simulation for a photon energy of 10 keV. (**a**) FEL gain curve. Spectral plots after the (**b**) first, (**c**) second, and (**d**) fifth undulator. The black-colored plots correspond to $\phi =0$ (the in-phase), and the blue and red plots indicate the out-of-phase $\phi =\pi$ and $1.15\pi$ results, respectively.

To analyze the two-color spectrum, we performed 1D time-dependent FEL simulations (a 1D case was assumed to eliminate other unwanted effects on the spectrum). We set a flat-top density profile of the electron beam without any wakefield and undulator tapering. The simulation parameters were based on the undulator parameters of the PAL-XFEL^20^, i.e., an undulator parameter K of 1.87, an undulator length of ${\mathrm{L}}_{\mathrm{u}}\hspace{0.17em}$= 5 m, the intersection length between the undulators was 1 m, an undulator wavelength of ${\uplambda }_{\mathrm{u}}\hspace{0.17em}$= 2.6 cm, a flat-top electron beam length of 20 µm, a beam current of 3000 A, a normalized beam emittance of 0.5 mm∙mrad, a beam energy spread of 0.01%, and a beam energy of 8.543 GeV corresponding to a photon energy of 10 keV. Phase shifters, which shift the phase $\phi$ of the electrons by a given value, were defined at all the intersections. We assumed that phase-shifting occurred only when the electrons travelled to the phase shifter positions and compared three cases, viz. $\phi =0$ (in-phase), $\uppi$ (out-of-phase), and 1.15 $\uppi$ (Fig. 3). We confirmed that although the phase shifters were set to be out-of-phase, the amplification of the FEL energy followed a log scale as shown in Fig. 3a. The spectra of the three cases were compared after the (b) first undulator, (c) second undulator, and (d) fifth undulator (just before the saturation). As expected, a two-color spectrum was observed at $\phi =\pi$ (blue-colored plots), whereas a balanced two-color spectrum was empirically obtained at $\phi =1.15 \pi$ (red-colored plots).

**Figure 4:**
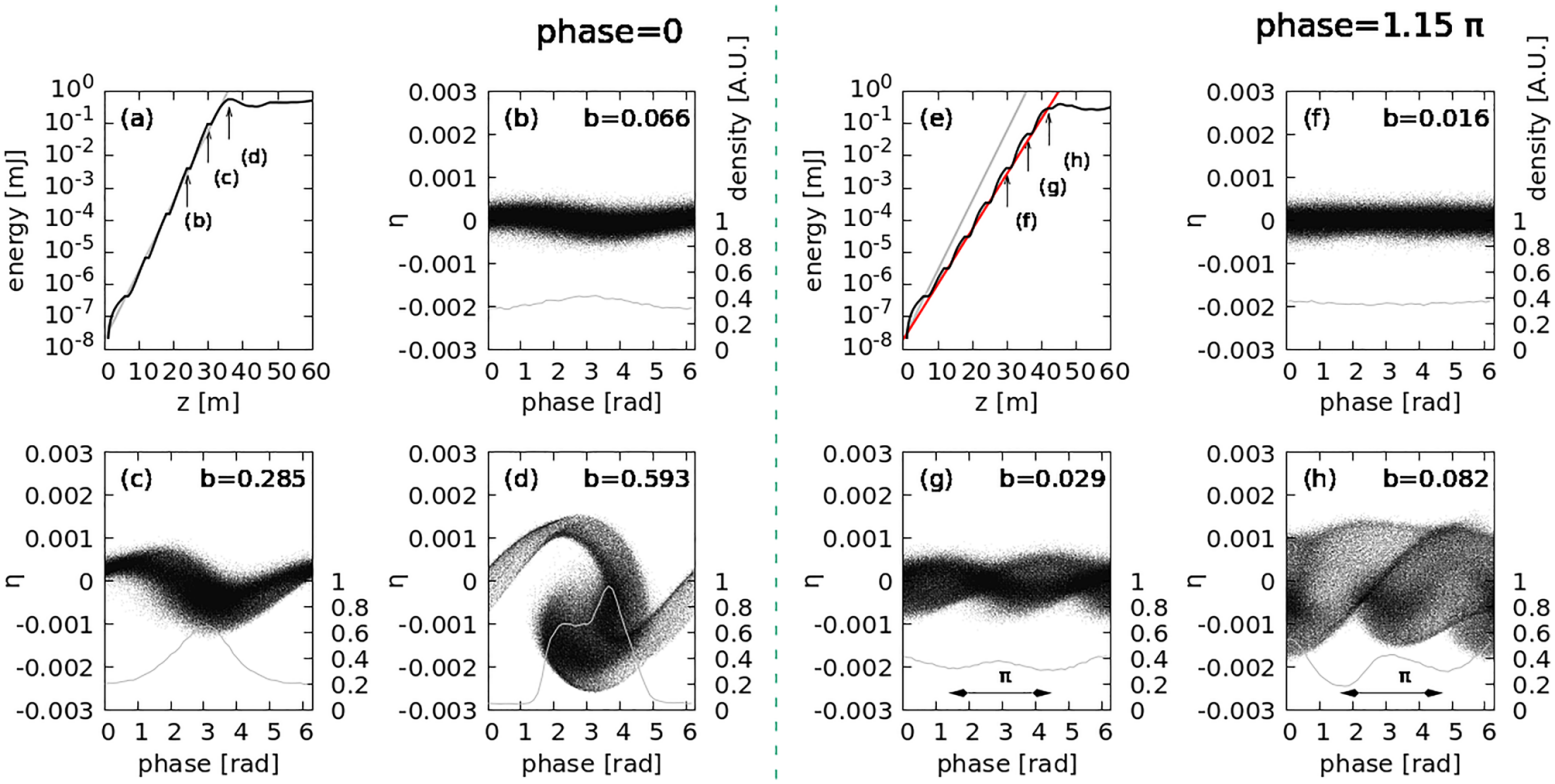
Comparison of in-phase and out-of-phase results. (**a**), (**e**) FEL gain curves; the gray line in (**a**) represents the fitting line, which is also plotted in (**e**). The red line in (**e**) is the fitting line for the out-of-phase setting, which indicates a line slope (0.793) that is smaller than that of the gray line. (**b**)–(**d**) and (**f**)–(**h**) Evolution of electron bunching; the gray line in each figure represents the density of electrons and the corresponding bunching factor is indicated. The out-of-phase setting results show a double bunching formation.

Amplification of two-color XFEL intensity was verified from the gain curve. The results obtained for two cases, viz. *ϕ* = 0 for a single-color spectrum and *ϕ* = 1.15 π for a balanced two-color spectrum, were compared. As the electron beam interacted with the FEL field, the progress of the electron bunches clearly indicated FEL amplification. To draw the electron phase space, electrons covering 50 slices around the beam center were collected (Fig. 4). The electron phase spaces and the density of electrons at various positions for the in-phase case are shown in Fig. 4(b)–(d). Corresponding bunching factors, $b=\langle {e}^{i\theta }\rangle$ where $\langle \dots \rangle$ is an average notation are also indicated. When the electrons accumulated around $\phi =\uppi$ (Fig. 4d), the FEL amplification ceased, and saturation was observed (Fig. 4a).

In the out-of-phase case, alternate phase-shifts were induced, i.e., y 0 to $\uppi$ or $\uppi$ to 0, at every undulator intersection. Unlike the case of $\phi =0$, for $\phi =1.15\uppi$ (same as $\phi =1\uppi$), two bunches were observed within one phase bucket, separated by approximately $\uppi$ (Fig. 4g,h). As a result, approximately 50% of the electrons around phase “π” were selected for FEL lasing, which affected the gain curve. As 50% of the electrons in the beam participates in FEL lasing, the beam current for the FEL lasing effectively reduces to half of its initial value. According to the relationship between FEL gain length and beam current (${L}_{g}\propto 1/\rho \propto 1/{I}^{1/3}$), the FEL gain length at 50% electron population elongated by approximately 26% compared to that at 100% electron population, and the FEL gain slope for the out-of-phase case decreased by 0.793. These results were confirmed by the 1D FEL simulations. The gain curve for the in-phase condition was fitted with the function $\mathrm{exp}\left(0.5z\right)$ (the gray line in Fig. 4a,e), and the expected gain curve was $\propto \mathrm{exp}\left(0.4z\right)$, consistent with the simulation result shown by the red line in Fig. 4e.

Note that the bunching factor of the out-of-phase condition does not significantly increase because the two bunches are developed within one phase bucket and are separated by $\pi$. In this case, $\langle {e}^{i\theta }\rangle$ term of the bunching factor still has a value close to zero even though 50% of the electrons are gathered and participate in the FEL amplification process. Therefore, the bunching factor is not suitable for analyzing the FEL amplification of the out-of-phase case, because the bunching factor is defined at the resonant frequency which is suppressed in our scheme.

**Figure 5:**
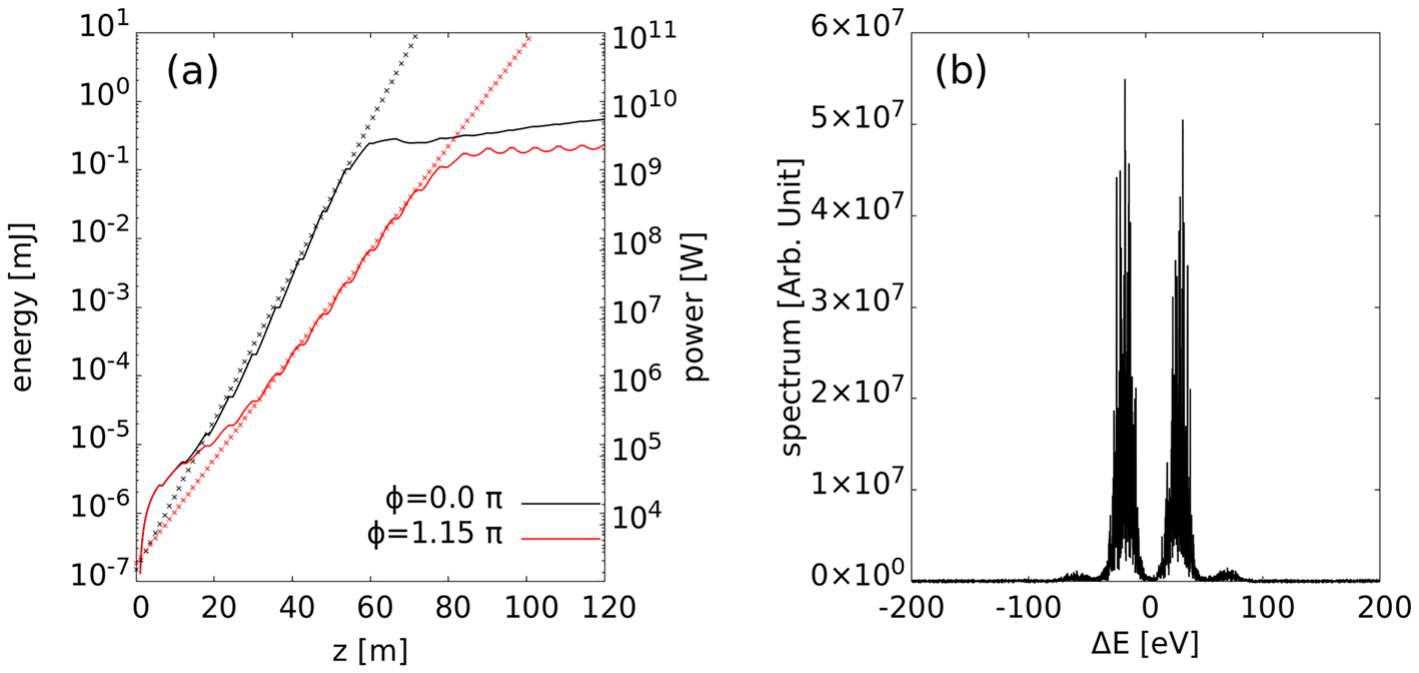
3D simulation results. (**a**) FEL gain curves of in-phase ($\phi =0.0 \pi$; black lines) and out-of-phase ($\phi =1.15 \pi$; red lines) cases. The dotted line represents the fitted line of gain curve in the linear regime. The black dotted line follows $\propto \mathrm{exp}\left(0.25z\right)$, and the red dotted line follows $\propto \mathrm{exp}\left(0.25\times 0.7z\right)$. (**b**) Two-color spectrum for the out-of-phase condition at $z=75 m$.

Further, the 3D simulation was carried out by using GENESIS code^21^ and the results (Fig. 5) were similar to the 1D results, except for the gain slope, whose fitted value was 0.7 in the 3D simulation case (0.793 for 1D). This discrepancy in the slope value can be attributed to the 3D effects of the field diffraction and evolution of the electron beam size. In the simulation, β matching was performed within a Twiss β parameter range of 10 to 20 m. Other features of the two-color spectrum were consistent with those from 1D simulation results.

## Experimental results

**Figure 6:**
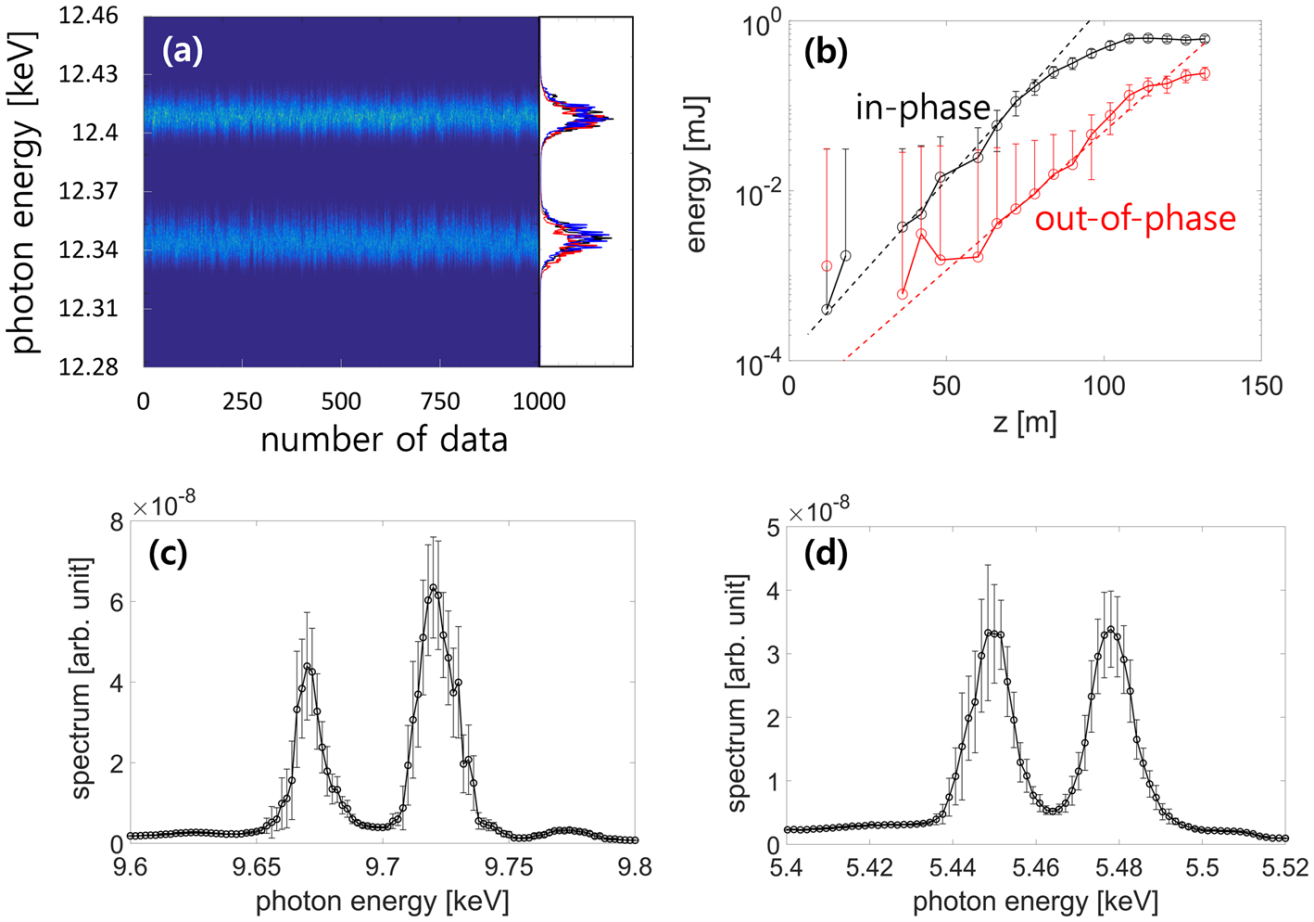
Experimental results of two-color XFEL pulses for different FEL photon energies. (**a**) Inline spectrometer measurements of out-of-phase condition for 1000 shots with three randomly selected plots of a single-shot spectrum and (**b**) the gain curves for in-phase (black lines) and out-of-phase (red lines) conditions at 12.38 keV (bars, ± 1 standard deviation (s.d.); number of measurements for each point (n) is 60). The dotted line represents the fitted line of gain curve in the linear regime. Spectrums of two-color XFEL pulses at out-of-phase condition for other photon energies were measured at (**c**) 9.7 keV and (**d**) 5.46 keV by using a photodiode after the double crystal monochromator (bars, ± 1 s.d.; n = 60).

We generated two-color XFEL pulses at the PAL-XFEL by setting the phase shifters as out-of-phase condition ($\phi =1.34 \pi$, see ‘Method’ section); the corresponding two-color spectrum is shown in Fig. 6. Figure 6a shows a collection of single-shot spectra obtained at 12.37 keV (the inline spectrometer uses a curved Si crystal). The measured energy difference of the two-color spectrum is 61.25 eV, which is close to the expected value of 64.37 eV (derived from Eq. (11)). Similar to the simulated results discussed in the previous section, the experimental slope of the FEL gain curve in the linear regime ($\mathrm{exp}\left(0.0714\times 0.8z\right)$) decreased to 80% of that obtained in the in-phase case ($\mathrm{exp}\left(0.0714z\right)$), as shown in Fig. 6b. A linear undulator taper was used in the experiment, and the intensity of the two-color FEL pulse was 250 µJ, whereas that observed the in-phase case was 590 µJ. For other photon energies of 9.7 and 5.46 keV (Fig. 6c,d), the measured energy differences were 50.4 and 28.4 eV, respectively, which were consistent with the simulated values.

## Summary

In this study, we developed a time-synchronized two-color XFEL pulse generation method that can be easily implemented by adjusting the gaps of phase shifters with a given undulator tapering. The small gain theory provides a theoretical description of the two-color seed; the energy separation in the two-color spectrum is determined by the number of undulator periods. Even when the phase setting is close to the FEL suppression condition, a relatively strong two-color FEL intensity can still be obtained. Further studies on undulator tapering and optimal phase setting for individual phase shifters may be necessary to achieve a highly optimized FEL intensity. Although the time-delay between the two-color pulses cannot be controlled in this scheme for usual pump-probe experiments, these time-synchronized two-color XFEL pulses are still an attractive option for various applications such as multiple wavelength anomalous dispersion and multicolor imaging. This can help mitigate the drawbacks of time-split two-color XFEL pulses.

## Methods

For FEL simulations, we used a home-made FEL code for 1D simulation and the GENESIS code^21^ for 3D simulation. Most of the parameters of the electron beam and undulators followed the PAL-XFEL specifications, except for the self-seeding section, which was ignored to exclude the drift effect of the self-seeding section. FEL simulations were performed in time-dependent mode to calculate the FEL spectrum. In 1 m long intersections, artificial undulators were inserted to match the phases between the electron beam and FEL. Phase shifters were activated at the center of every intersection. The major simulation parameters are summarized in Table 1.

**Table 1:** Parameters used in simulations and experiments.

Undulator length	5 m
Intersection length	1 m
Undulator K	1.87
Number of undulator units	21
Electron beam current	3000 A
Electron beam charge	200 pC
Electron beam emittance	0.5 mm mrad
Electron beam shape	Flat-top (simulation)
Photon energy	10 keV (simulation) 12.38, 9.7, 5.46 keV (experiment)

**Figure 7:**
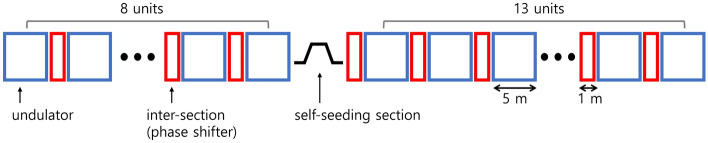
Layout of the hard X-ray undulator line at the PAL-XFEL.

**Figure 8:**
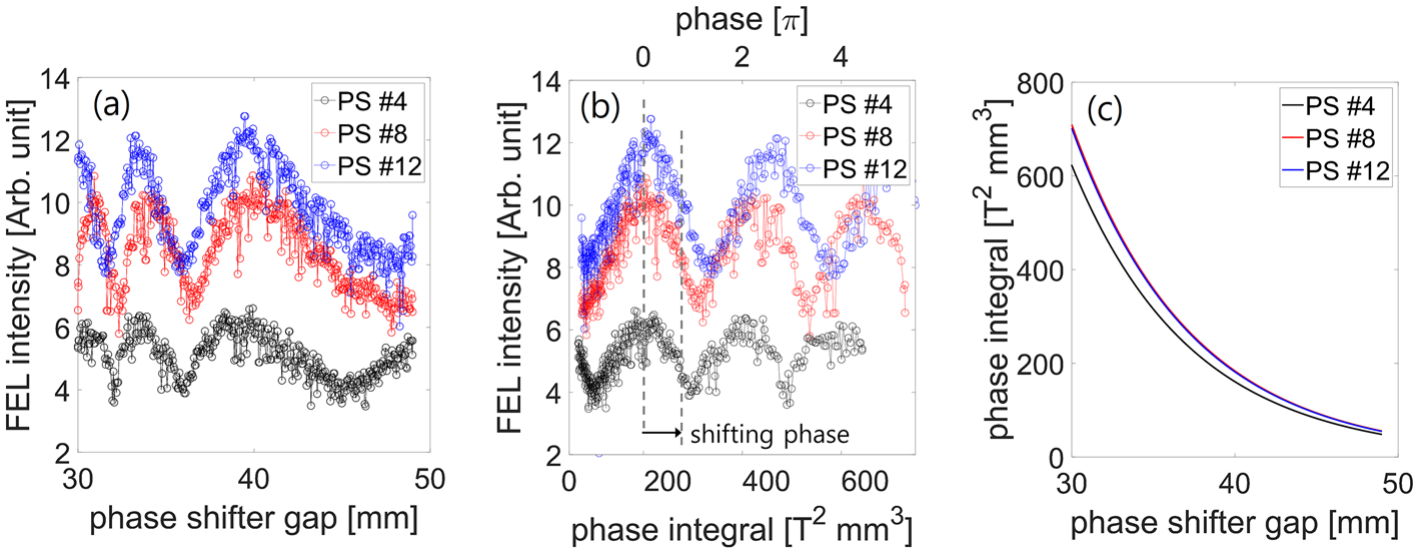
(**a**) Phase shifter scanning by changing the phase shifter gap. (**b**) Same as (a); the phase shifter gap was converted to the phase integral and the corresponding phase, which shows periodic changes. (**c**) Phase integral of the phase shifter with respect to the phase shifter gap for various phase shifters.

In experiments, the hard X-ray undulator line in the PAL-XFEL consists of 21 undulator segments, one self-seeding section, and 20 phase shifters between the undulator segments (Fig. 7). Each phase shifter can be considered as a mini-chicane, which delays the electron beam path. The phase shifter gap (which determines the phase shifter magnetic field) typically controls the delay length for several X-ray wavelengths. The delay length can be expressed as:

12 $$s=\frac{1}{2{\gamma }^{2}}\left({L}_{\mathrm{int}}+{\left(\frac{e}{mc}\right)}^{2}\cdot P{I}_{\mathrm{PS}}\right),$$

13 $$P{I}_{\mathrm{PS}}=\underset{-\infty }{\overset{\infty }{\int }}{\left(\underset{-\infty }{\overset{{z}^{{\prime}{\prime}}}{\int }}{B}_{\mathrm{y},\mathrm{PS}}\left({z}{\prime}\right)d{z}{\prime}\right)}^{2}d{z}^{{\prime}{\prime}},$$

where ${L}_{\mathrm{int}}$ is the intersection length, ${B}_{\mathrm{y},\mathrm{PS}}$ is the measured magnetic field strength of the phase shifter, $\gamma$ is the Lorentz factor of electron, and $P{I}_{\mathrm{PS}}$ is the phase integral. Examples of the phase shifter gap scan are shown in Fig. 8a. The delay length $s$ varies linearly with $P{I}_{\mathrm{PS}}$, and thus, it is convenient to plot the phase shifter scan data in terms of $P{I}_{\mathrm{PS}}$. The same result is shown in Fig. 8b after converting the phase shifter gap to $P{I}_{\mathrm{PS}}$ by using a measured table of $P{I}_{\mathrm{PS}}$ values according to the phase shifter gap (Fig. 8c). The out-of-phase condition can be obtained by setting all the phase shifters to the minimum FEL intensity while performing the phase shifter scan. However, obtaining a balanced two-color spectrum with all the phase shifters is a considerable challenge. In the experiment, a linear undulator taper was set, and the optimal FEL intensity was determined via phase shifter scans. The optimal condition (at the maximum FEL intensity) was assumed to be the in-phase condition. Then, the $P{I}_{\mathrm{PS}}$ s of all the phase shifters were changed by adding the same $\Delta P{I}_{\mathrm{PS}}$ value until a two-color spectrum with balanced peaks was observed from the inline spectrometer. For example, a balanced two-color XFEL spectrum was obtained by shifting from the in-phase setting by $\Delta P{I}_{\mathrm{PS}}=66$ (it was around 0.67 π (or 1.34 π)); at this position, the FEL intensity was not minimum (Fig. 8b). Our simulation results indicate that the condition 1.15 π is optimal for obtaining a balanced spectrum. However, the phase-shifter setting in experiments can be varied depending on the electron beam parameters and undulator settings.

## Data Availability

The datasets used and analyzed during the current study available from the corresponding author on reasonable request.
